# Supplementary material for: Dysregulation of M segment gene expression contributes to influenza A virus host restriction
Source: PLoS Pathog. 2019 Aug 15;15(8):e1007892. doi: 10.1371/journal.ppat.1007892 (PMC6695095; doi:10.1371/journal.ppat.1007892)
Supplement: S1 Fig — (A) The M segment of influenza virus is template for synthesis of mRNA7 (encoding M1), mRNA M10 (encoding M2), and mRNA11 (which encodes a putative but unconfirmed 10 amino acid peptide from a short open reading frame). (B) Pandemic H1N1 influenza virus M1 and M2 proteins differ from the avian consensus sequences by 9 residues in M1 and 7 residues in M2. The M segments differ by 8.3% at the nucleotide level. (C) Seasonal H3N2 influenza virus strain A/Panama/2007/99 M1 and M2 proteins differ from the avian consensus sequences by 11 residues in M1 and 14 residues in M2. The M segments differ by 9% at the nucleotide level. (D) Seasonal H3N2 influenza virus strain A/Bethesda/55/15 M1 and M2 proteins differ from the avian consensus sequences by 11 residues in M1 and 16 residues in M2. The M segments differ by 9.5% at the nucleotide level. (PDF) [file ppat.1007892.s001.pdf]

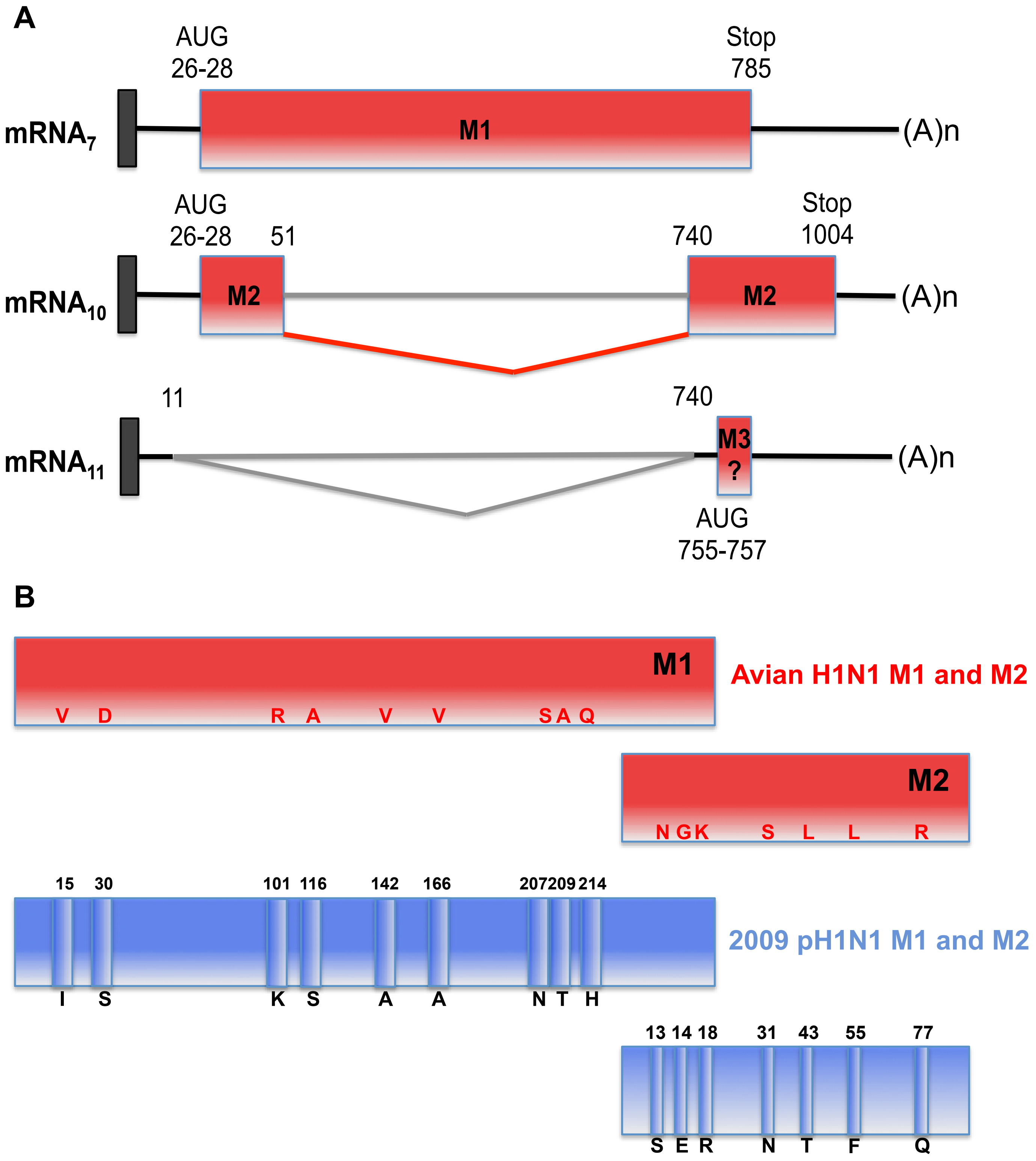
A
AUG
26-28
Stop
785
mRNA7
M1
(A)n
AUG
26-28
51
740
Stop
1004
mRNA10
M2
M2
(A)n
11
740
mRNA11
M3
?
(A)n
AUG
755-757
B
M1
V D R A V V S A Q
Avian H1N1 M1 and M2
M2
N G K S L L R
15 30 101 116 142 166 207 209 214
I S K S A A N T H
2009 pH1N1 M1 and M2
13 14 18 31 43 55 77
S E R N T F Q

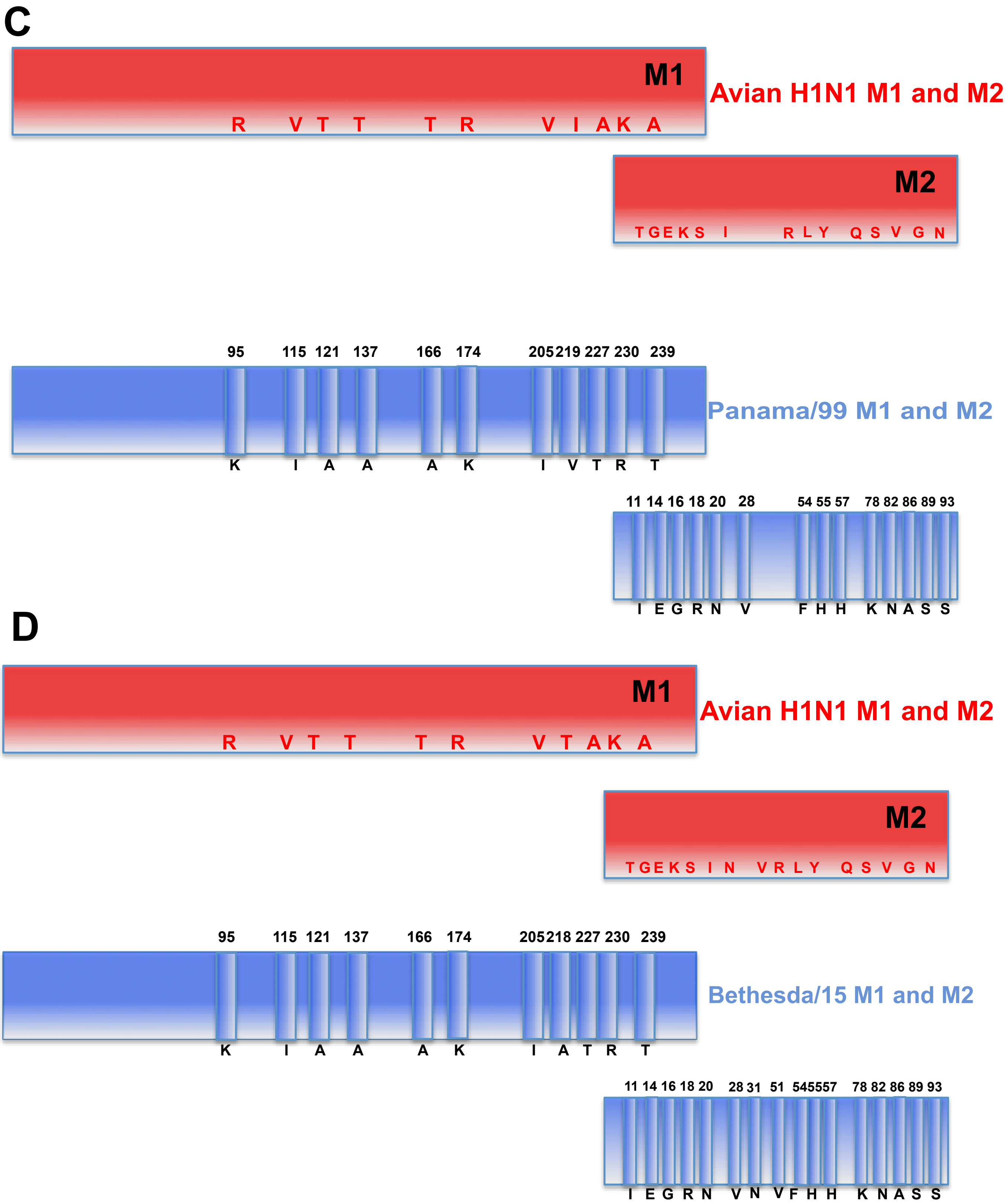

Supplementary Figure 1

Supplementary Figure 1. Schematic of M segment mRNAs and gene products

A. The M segment of influenza virus is template for synthesis of $mRNA_7$ (encoding M1), mRNA M_{10} (encoding M2), and $mRNA_{11}$ (which encodes a putative but unconfirmed 10 amino acid peptide from a short open reading frame). **B.** Pandemic H1N1 influenza virus M1 and M2 proteins differ from the avian consensus sequences by 9 residues in M1 and 7 residues in M2. The M segments differ by 8.3% at the nucleotide level. **C.** Seasonal H3N2 influenza virus strain A/Panama/ 2007/99 M1 and M2 proteins differ from the avian consensus sequences by 11 residues in M1 and 14 residues in M2. The M segments differ by 9% at the nucleotide level. **D.** Seasonal H3N2 influenza virus strain A/Bethesda/55/15 M1 and M2 proteins differ from the avian consensus sequences by 11 residues in M1 and 16 residues in M2. The M segments differ by 9.5% at the nucleotide level.
