# Supplementary material for: Dysregulation of M segment gene expression contributes to influenza A virus host restriction
Source: PLoS Pathog. 2019 Aug 15;15(8):e1007892. doi: 10.1371/journal.ppat.1007892 (PMC6695095; doi:10.1371/journal.ppat.1007892)
Supplement: S2 Fig — PR8-based viruses were inoculated at a MOI of 5 PFU/cell onto human-derived 293T cells (A), or A549 cells (B). Cells were incubated at 37°C for up to 24 h. Virus released into supernatant was collected at the indicated time points, and virus growth was measured by plaque titration. Data obtained from viruses possessing human M segments are represented with blue lines: A/NL/602/09 M (A,B), A/Panama/2007/99 M (B), and A/Bethesda/15 M (B), while data from viruses encoding avian M segments are represented with red lines. In each cell type, the human M segments conferred more rapid kinetics and higher peak titers of growth than any avian-origin M segment. Single-cycle growth was assessed in three independent experiments, with three technical sample replicates per experiment. Graphs show the means with SD for the three experiments. Statistical significance was determined using repeated measures, two-way, multiple ANOVA on log-transformed data, with Bonferroni correction applied as there were a limited no of means to compare. (PDF) [file ppat.1007892.s002.pdf]

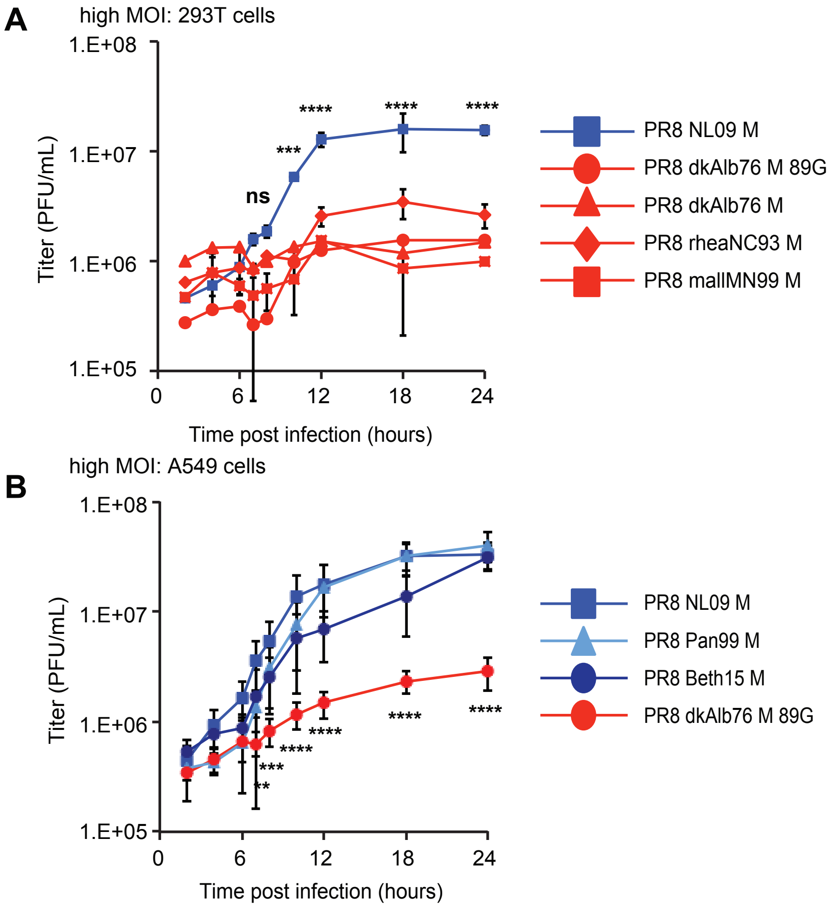

Supplementary Figure 2. Human Host-derived M Segments Confer Higher Growth to PR8-Based Viruses than Avian Host-derived M Segments in Mammalian Cells at 37 °C.

PR8-based viruses were inoculated at an MOI of 5 onto human-derived 293T cells **(A)**, or A549 cells **(B)**. Cells were incubated at 37°C for up to 24 hours. Virus released into supernatant was collected at the indicated time-points, and virus growth was measured by plaque titration. Data obtained from viruses possessing human M segments are represented with blue lines: A/NL/602/09 M (**A,B**), A/Panama/2007/99 M (**B**), and A/Bethesda/15 M (**B**), while data from viruses encoding avian M segments are represented with red lines. In each cell type, the human M segments conferred more rapid kinetics and higher peak titers of growth than any avian-origin M segment. Single-cycle growth was assessed in three independent experiments, with three technical sample replicates per experiment. Graphs show the means with SD for the three experiments. Statistical significance was determined using repeated measures, two way, multiple ANOVA on log transformed data, with Bonferroni correction applied as there were a limited no of means to compare.
