# Supplementary material for: Dysregulation of M segment gene expression contributes to influenza A virus host restriction
Source: PLoS Pathog. 2019 Aug 15;15(8):e1007892. doi: 10.1371/journal.ppat.1007892 (PMC6695095; doi:10.1371/journal.ppat.1007892)
Supplement: S4 Fig — 293T and MDCK cells were inoculated at a MOI of 5 PFU/cell with PR8 viruses encoding avian or human-derived M segments and incubated at 37°C for 8 h, then cells were lysed. Western immunoblot analysis of virus-infected 293T cells (A) and MDCK cells (G). Vinculin expression was measured to allow normalization of viral protein levels. NP expression was measured to assess viral replication. Levels of M1 and M2 protein expression were assessed using an antibody (Mab E10) to a common epitope at the amino terminus of M1 and M2 proteins, allowing relative expression to be assessed. Levels of LC3B I and II were assessed using an antibody that detects both the precursor and activated forms of LC3B protein. (B, H) M1 protein and (C, I) M2 protein were normalized to vinculin, quantitated and displayed as a percentage of total protein expressed from the M gene. (D, J) The ratio of M1:M2 protein expression. (E, K) LC3B I protein and (F, L) LC3BII protein were normalized, quantitated and displayed as a percentage of total LC3B protein. Graphs in B-F, and H-K show the means with SD from three independent experiments. For each experiment, two replicate Western immunoblots were performed and quantitated. Statistical significance was assessed using ordinary one-way ANOVA. (PDF) [file ppat.1007892.s004.pdf]

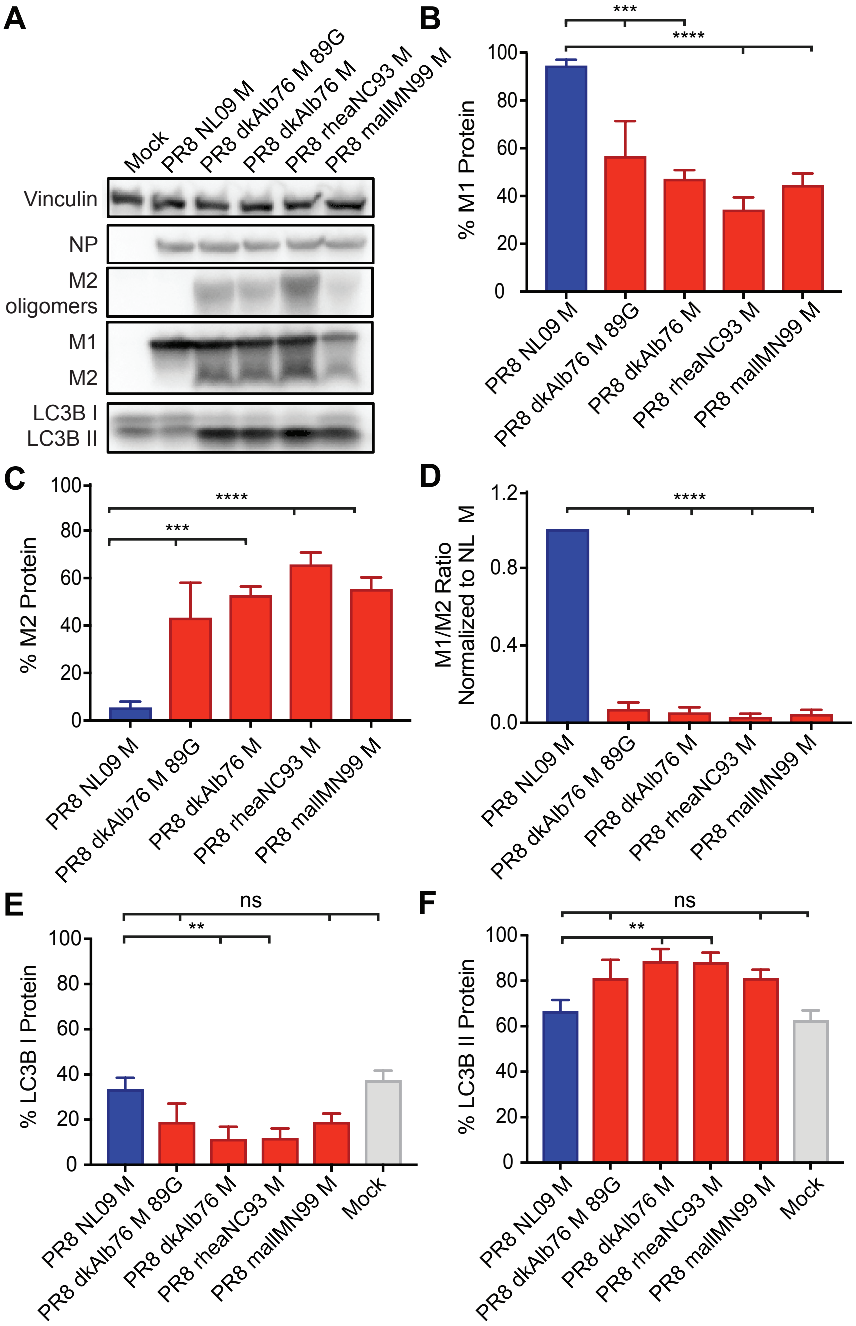

A
Mock
PR8 NL09 M
PR8 dkAlb76 M 89G
PR8 dkAlb76 M
PR8 rheaNC93 M
PR8 mallMN99 M
Vinculin
NP
M2
oligomers
M1
M2
LC3B I
LC3B II
B
% M1 Protein
***
****
C
% M2 Protein
***
****
D
M1/M2 Ratio
Normalized to NL M
****
E
% LC3B I Protein
ns
**
F
% LC3B II Protein
ns
**

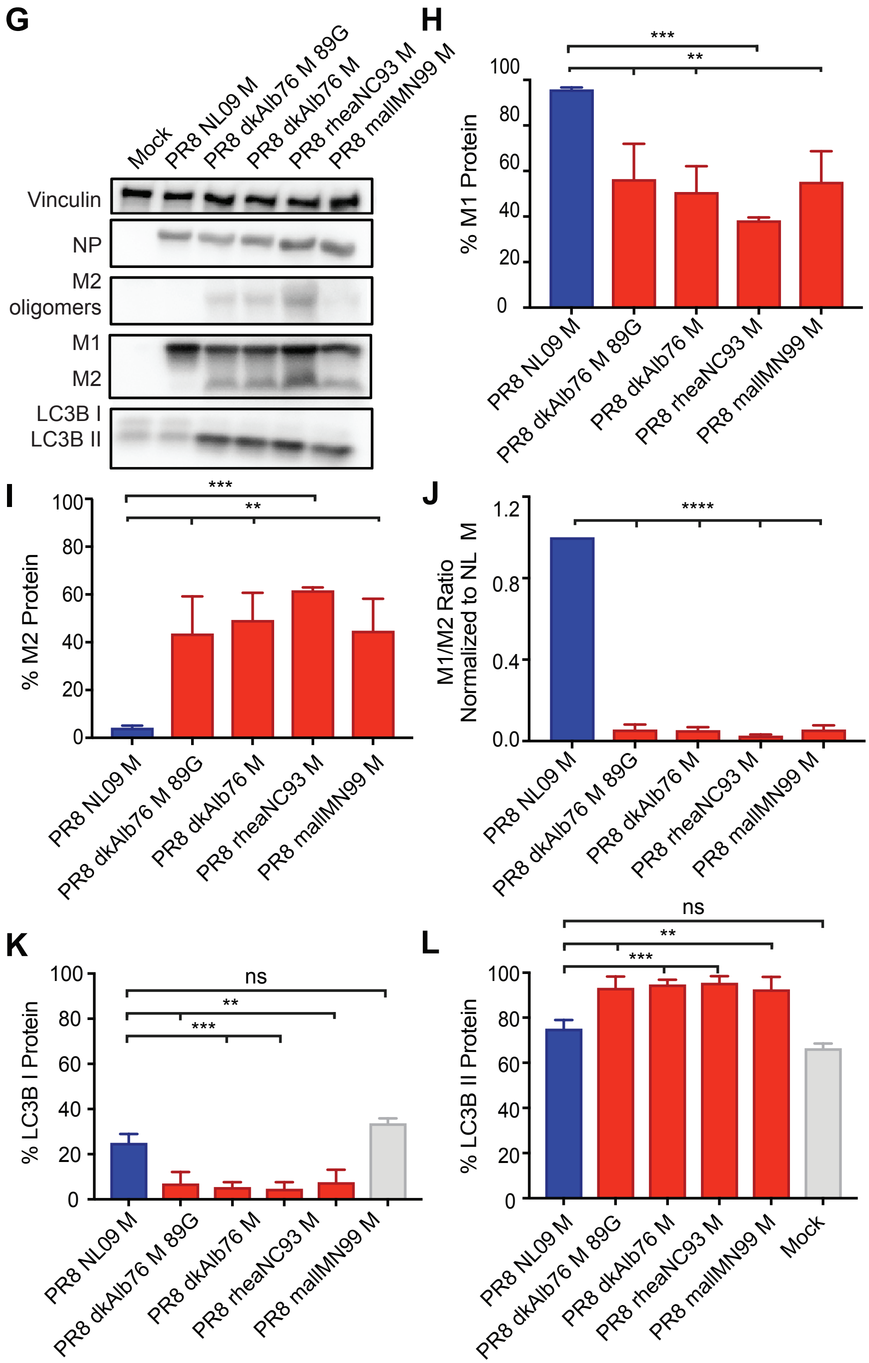


**Supplementary Figure 4. High Expression Ratio of M1 to M2 Protein in Human Cells is Dependent upon Viral M Segment Host Origin.**

293T and MDCK cells were inoculated at MOI=5 with PR8 viruses possessing M segments from human or avian host-derived strains and incubated at 37° C for 8 hours, then cells were lysed with whole cell lysis buffer. Western blot analysis of virus-infected 293T cells **(A)** and MDCK cells **(G)**. Vinculin expression was measured to allow normalization of viral protein levels. NP expression was measured to assess viral replication. Levels of M1 and M2 protein expression were assessed using an antibody (Mab E10) to a common epitope at the amino terminus of M1 and M2 proteins, allowing relative expression to be assessed. Levels of LC3B I and II were assessed using an antibody that detects both the precursor and activated forms of LC3B protein. M1 protein **(B, H)** and M2 protein **(C, I)** were normalized to vinculin, quantitated and displayed as a percentage of total protein expressed from the M gene. The ratio of M1:M2 protein expression was calculated and is plotted in **(D, J)**. LC3B I protein **(E, K)** and LC3B II protein **(F, L)** were normalized, quantitated and displayed as a percentage of total LC3B protein. Graphs in **B-F**, and **H-K** show the means with SD from three independent experiments. For each experiment, two replicate Western blots were performed and quantitated. Statistical significance was assessed using ordinary one-way ANOVA.
