# Supplementary material for: Dysregulation of M segment gene expression contributes to influenza A virus host restriction
Source: PLoS Pathog. 2019 Aug 15;15(8):e1007892. doi: 10.1371/journal.ppat.1007892 (PMC6695095; doi:10.1371/journal.ppat.1007892)
Supplement: S5 Fig — A549 cells were inoculated at a MOI of 5 PFU/cell with PR8 viruses encoding avian or human-derived M segments and incubated at 37°C for 8 h, then cells were lysed. Human M segments were derived from the following viruses: A/NL/602/09 (H1N1) (NL09); A/Panama/2007/99 (H3N2) (Pan99); and A/Bethesda/15 (H3N2) (Beth15). Vinculin expression was measured to allow normalization of viral protein levels. NP expression was measured to assess viral replication. Levels of M1 and M2 protein expression were assessed using an antibody (Mab E10) to a common epitope at the amino terminus of M1 and M2 proteins, allowing relative expression to be assessed. Levels of LC3B I and II were assessed using an antibody that detects both the precursor and the activated forms of LC3B protein. Data presented are representative of Western immunoblots from three independent experiments. (PDF) [file ppat.1007892.s005.pdf]

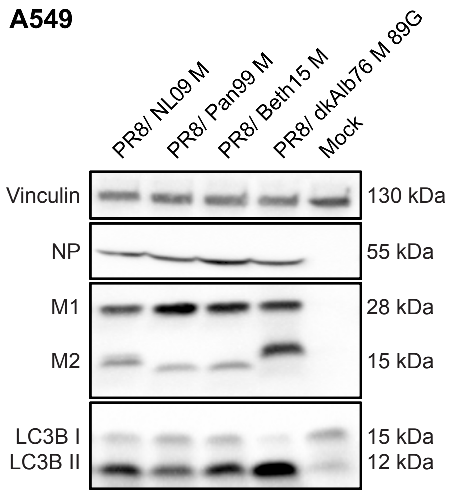

Supplementary Figure 5. High Expression Ratio of M1 to M2 protein in Human Cells is Dependent upon Viral M Segment Host Origin.

A549 cells were inoculated at MOI=5 with PR8 viruses possessing M segments from human or avian host-derived strains and incubated at 37º C for 8 hours, then cells were lysed with whole cell lysis buffer. Human M segments were derived from the following viruses: A/NL/602/09N (H1N1) (NL09); A/Panama/2007/99 (H3N2) (Pan99); and A/Bethesda/15 (H3N2) (Beth15). Vinculin expression was measured to allow normalization of viral protein levels. NP expression was measured to assess viral replication. Levels of M1 and M2 protein expression were assessed using an antibody (Mab E10) to a common epitope at the amino terminus of M1 and M2 proteins, allowing relative expression to be assessed. Levels of LC3B I and II were assessed using an antibody that detects both the precursor and the activated forms of LC3B protein. Data presented are representative of Western blots from three independent experiments.
