# Supplementary material for: Dysregulation of M segment gene expression contributes to influenza A virus host restriction
Source: PLoS Pathog. 2019 Aug 15;15(8):e1007892. doi: 10.1371/journal.ppat.1007892 (PMC6695095; doi:10.1371/journal.ppat.1007892)

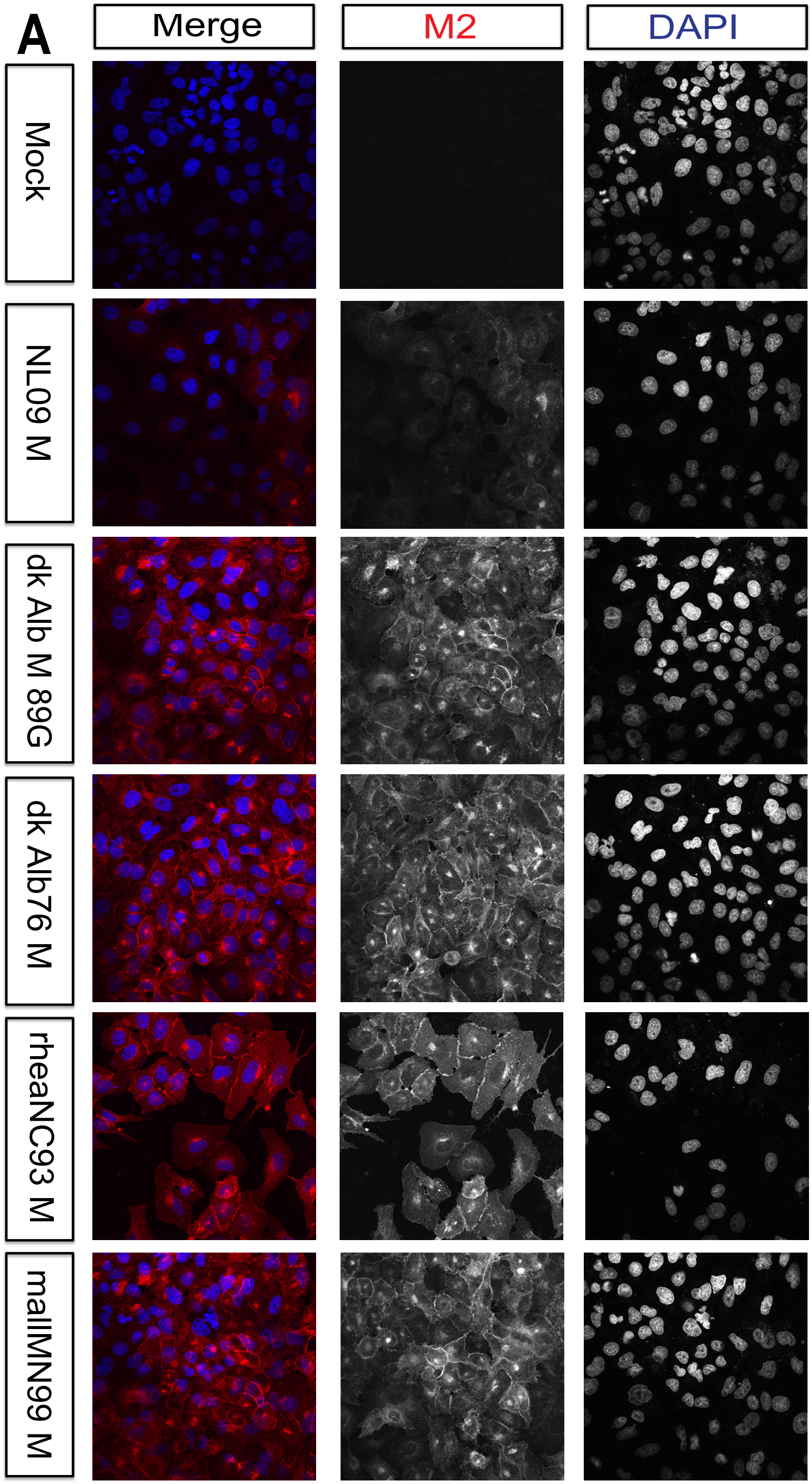
A
Merge
M2
DAPI
Mock
NL09 M
dk Alb M 89G
dk Alb76 M
rheaNC93 M
mallMN99 M

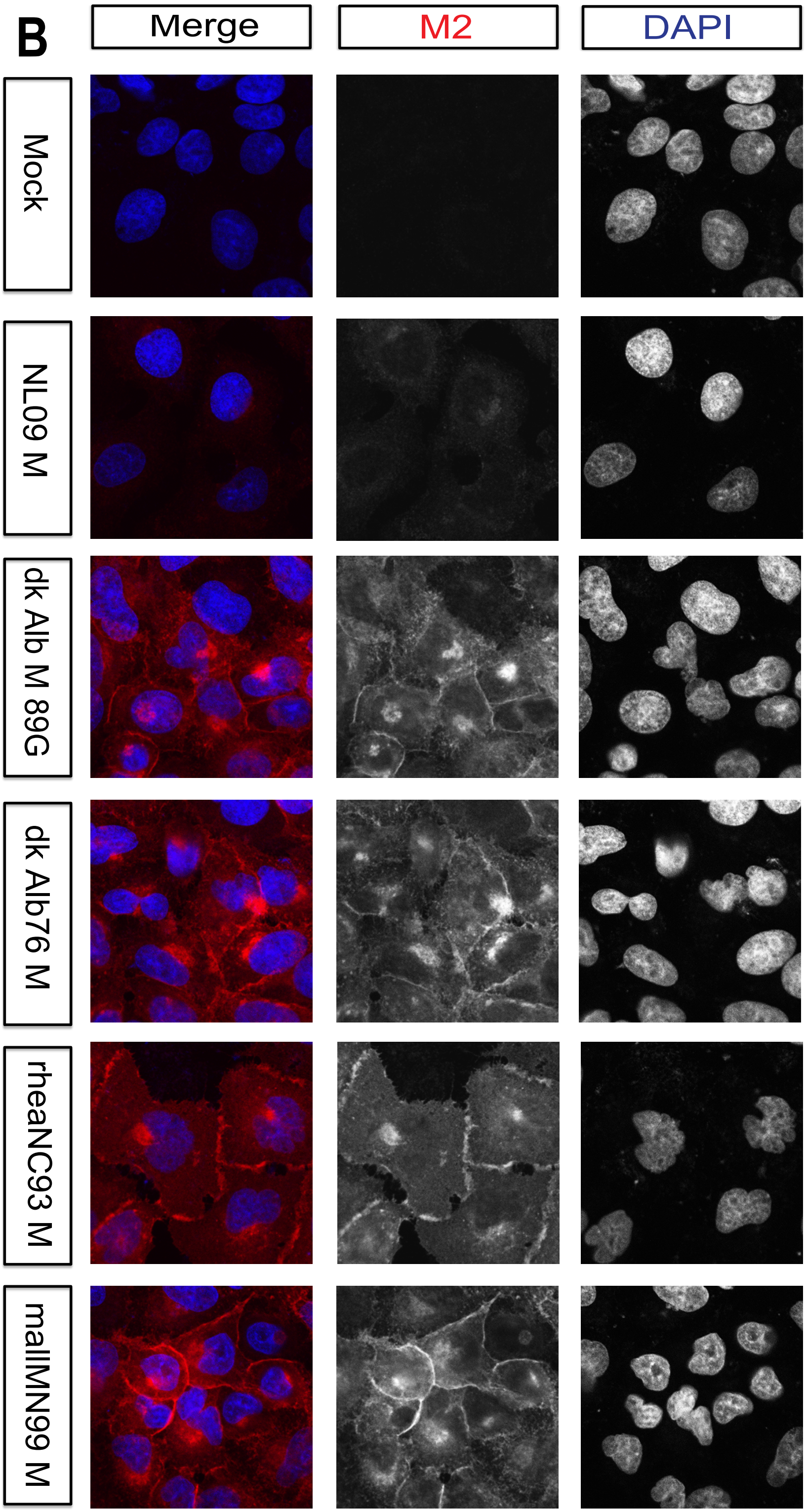
B
Merge
M2
DAPI
Mock
NL09 M
dk Alb M 89G
dk Alb76 M
rheaNC93 M
mallMN99 M

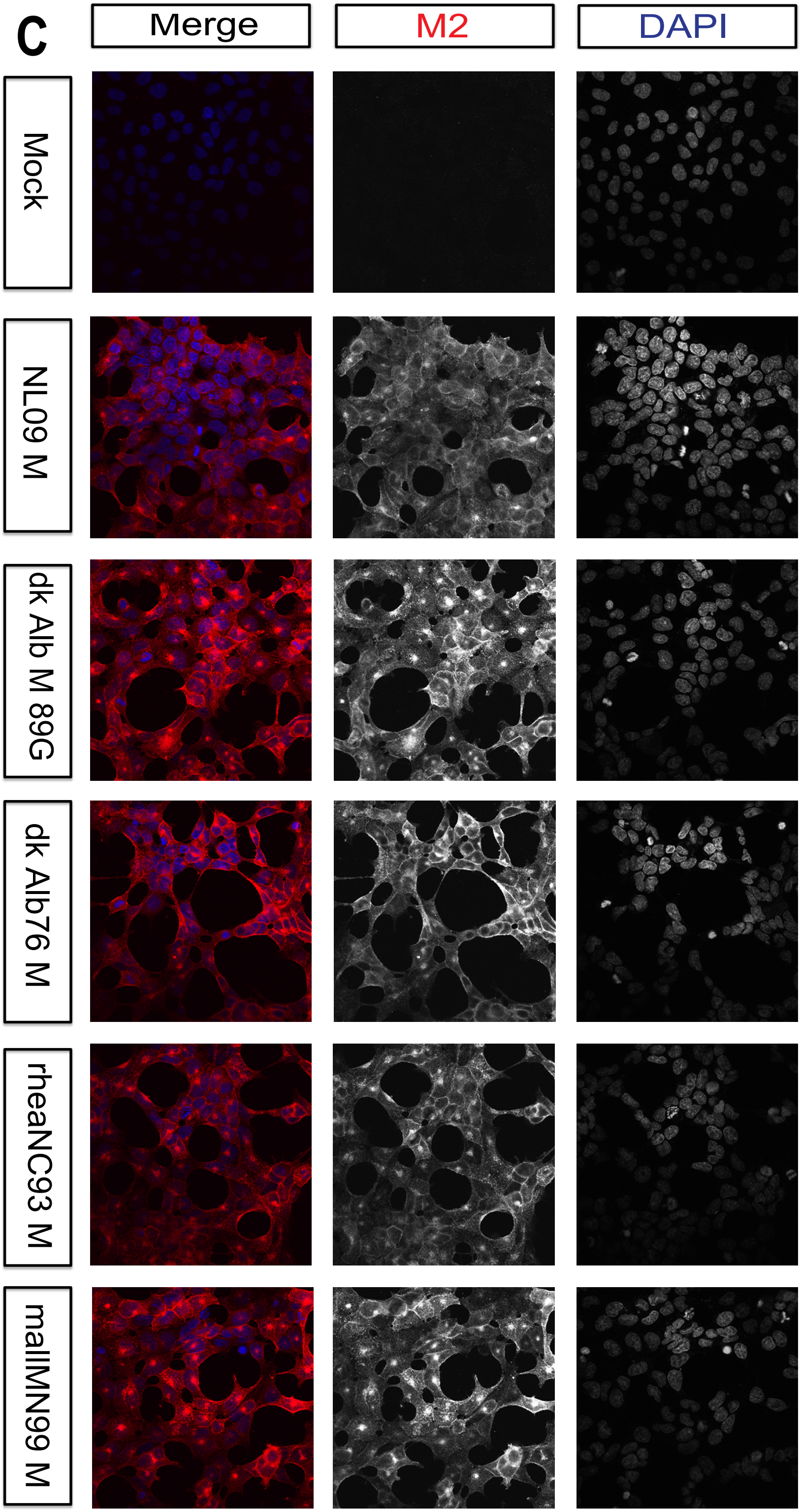

C
Merge
M2
DAPI
Mock
NL09 M
dk Alb M 89G
dk Alb76 M
rheaNC93 M
mallMN99 M

Supplementary Figure 6. Visualization of M2 localization by immunofluorescence microscopy at 12 h post-infection in A549, and 293T cells.

A549 (**A, B**), or 293T (**C, D**) cells were inoculated with the indicated viruses, encoding avian or human M segments, at an MOI of 5 PFU/cell. Cells were fixed at 12 h p.i., permeabilised, and stained with anti-M2 (Mab E10; red) and DAPI (blue) followed by imaging with confocal microscopy. Examples of optical sections are shown, either as merged 2-color images or the red and blue channels alone (in grey scale). **(A)** A549 cells with 63x magnification. **(B)** 3x magnification of the same images shown in **A**. **(C)** 293T cells with 63x magnification. **(D)** 3x magnification of the same images shown in **C**. **(D)** A549 cells with 63x magnification. **(E)** 3x magnification of the same images shown in **D**. Brightness was adjusted for optimal clarity, with all images treated equally.

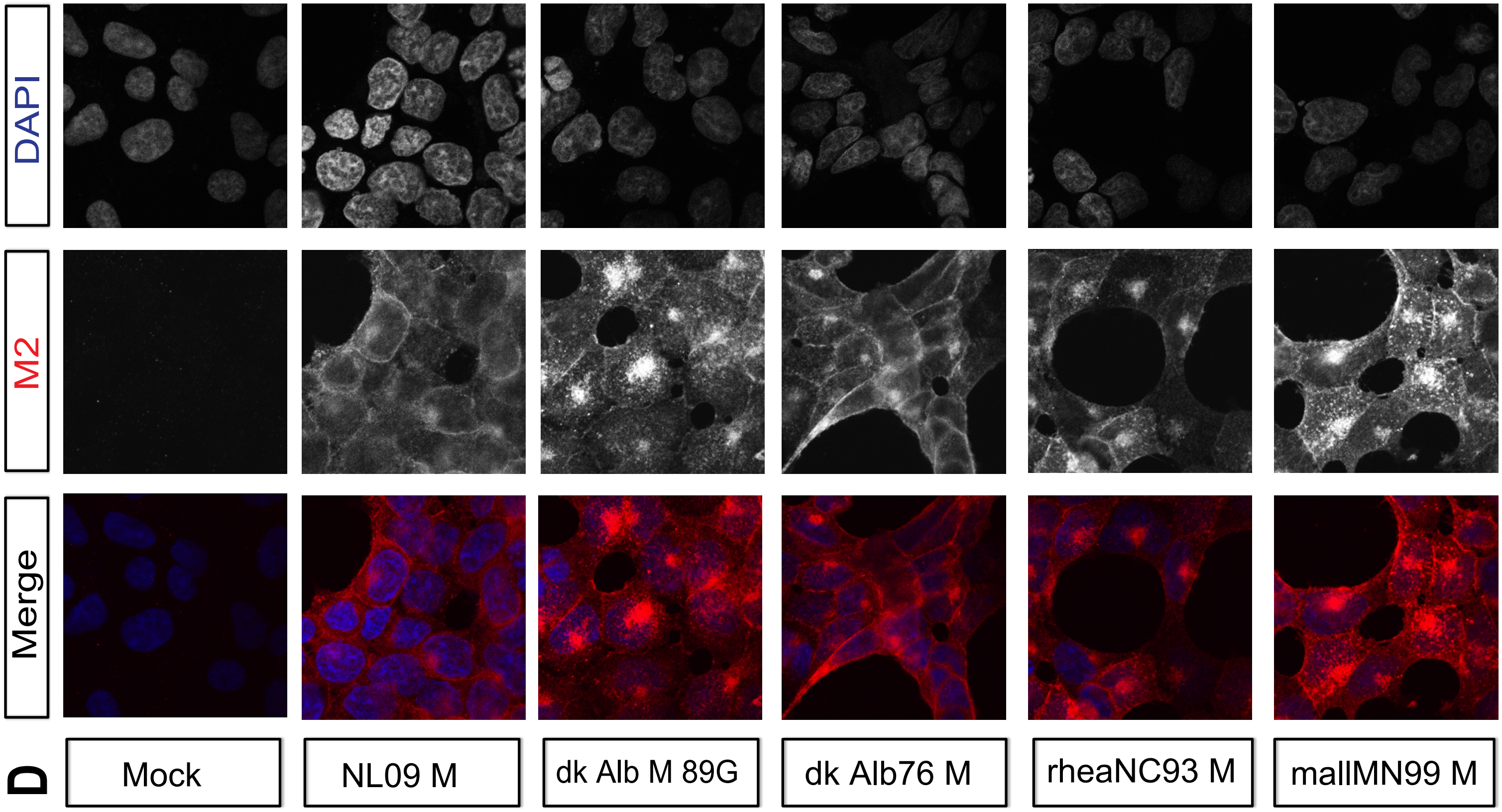

Supplement: S6 Fig — A549 (A, B), or 293T (C, D) cells were inoculated with the indicated viruses, encoding avian or human M segments, at a MOI of 5 PFU/cell. Cells were fixed at 12 hpi, permeabilised, and stained with anti-M2 (Mab E10; red) and DAPI (blue) followed by imaging with confocal microscopy. Examples of optical sections are shown, either as merged 2-color images or the red and blue channels alone (in grey scale). (A) A549 cells with 63x magnification. (B) 3x magnification of the same images shown in A. (C) 293T cells with 63x magnification. (D) 3x magnification of the same images shown in C. Brightness was adjusted for optimal clarity, with all images treated equally. (PDF) [file ppat.1007892.s006.pdf]
