# Supplementary material for: Dysregulation of M segment gene expression contributes to influenza A virus host restriction
Source: PLoS Pathog. 2019 Aug 15;15(8):e1007892. doi: 10.1371/journal.ppat.1007892 (PMC6695095; doi:10.1371/journal.ppat.1007892)

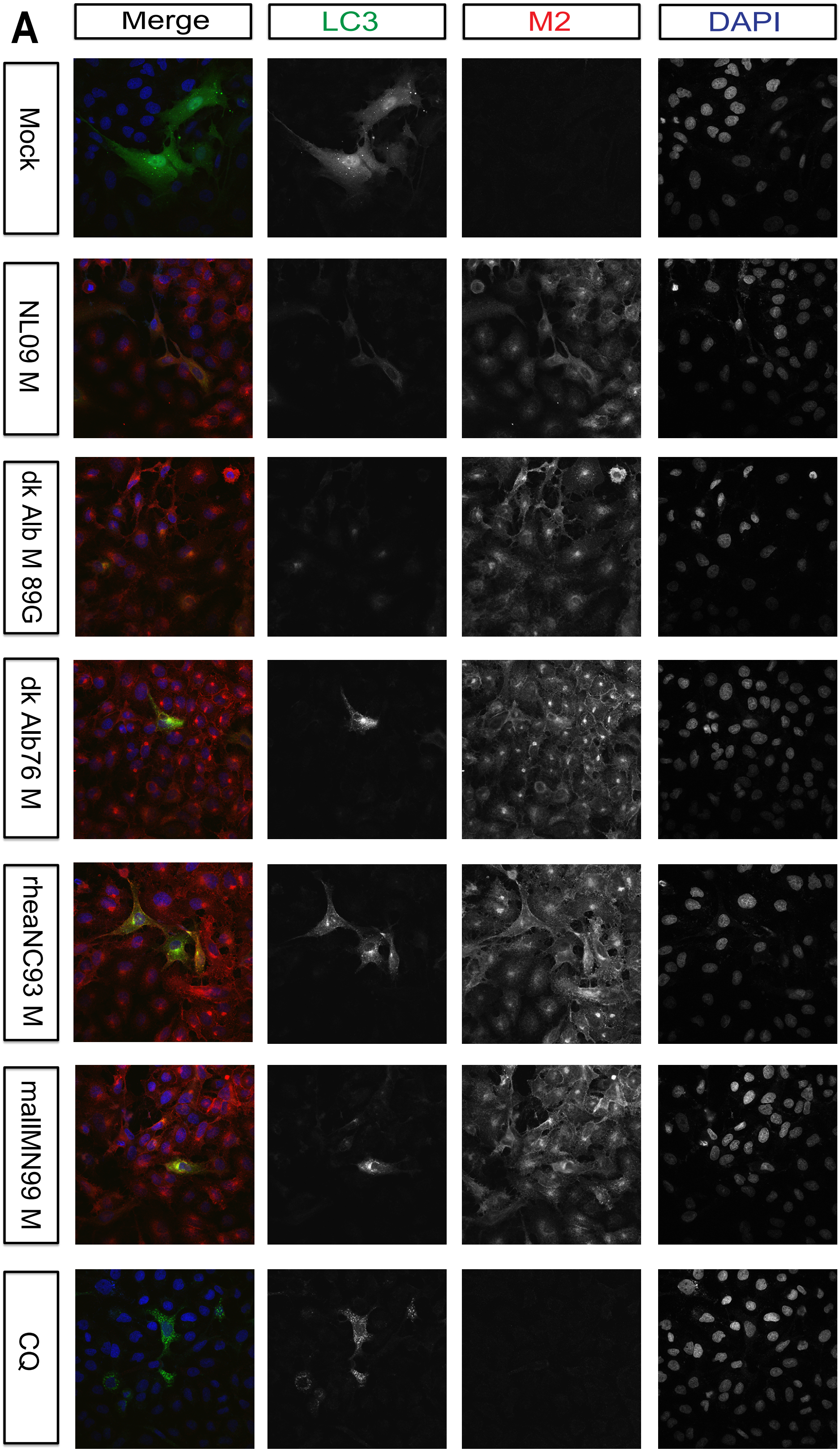
A
Merge
LC3
M2
DAPI
Mock
NL09 M
dk Alb M 89G
dk Alb76 M
rheaNC93 M
mallMN99 M
CQ

Supplementary Figure 7. Visualization of LC3 and M2 co-localization by immunofluorescence microscopy.

A549 cells were transduced with GFP-LC3 protein and inoculated 24 h later with the indicated IAVs, encoding avian or human derived M segments, at an MOI of 5 PFU/cell. Cells were fixed 8 h later and stained with anti-M2 (Mab E10; red) and DAPI (blue) followed by imaging with confocal microscopy. Examples of optical sections are shown, either as merged 3-color images or the red, green, and blue channels alone (in grey scale). **(A)** 63x magnification. **(B)** 3x magnification of the same images shown in **A**. Brightness was adjusted for optimal clarity, with all images treated equally.

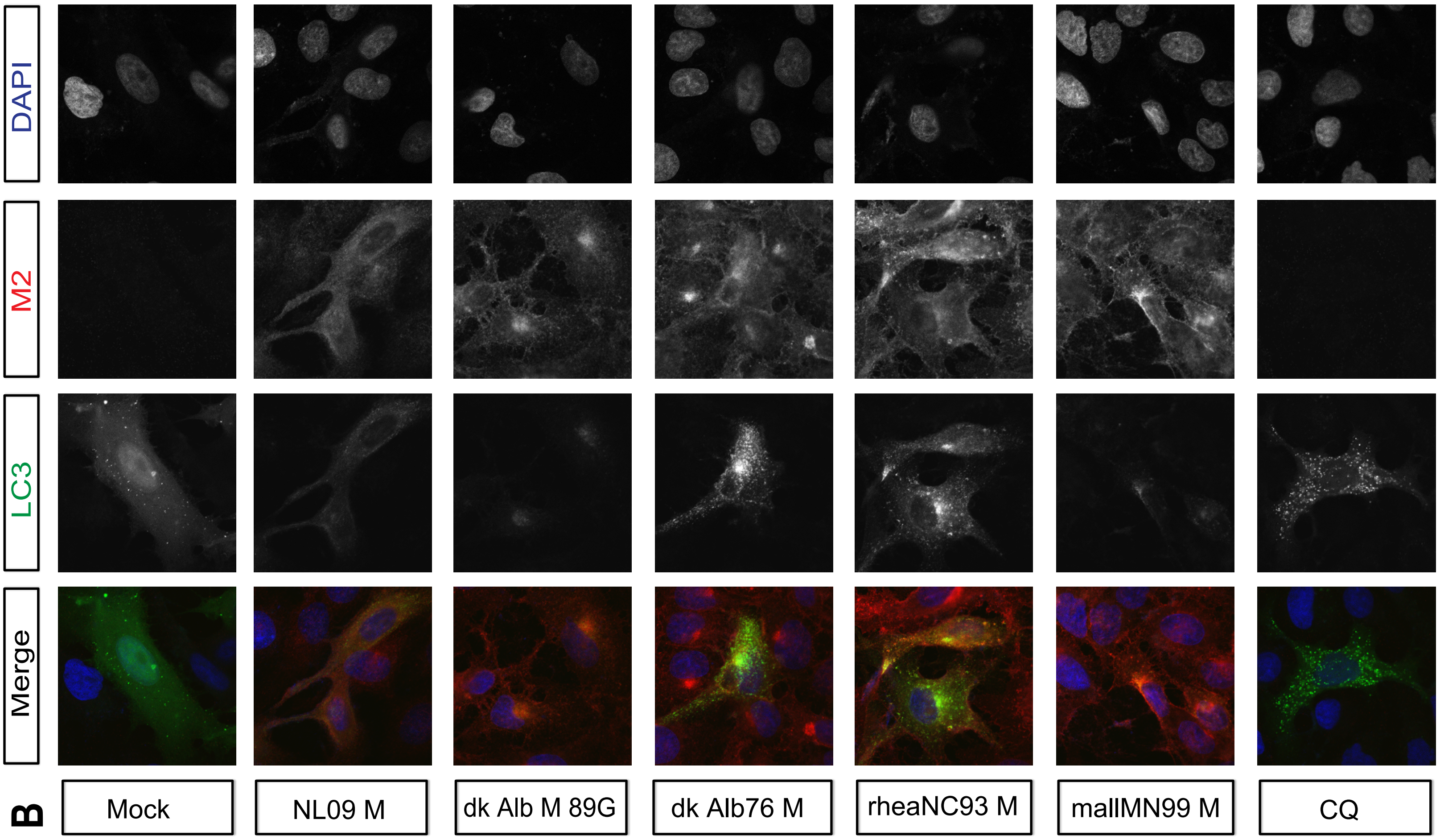

Supplement: S7 Fig — A549 cells were transduced with GFP-LC3 protein and inoculated 24 h later with the indicated IAVs, encoding avian- or human-derived M segments, at a MOI of 5 PFU/cell. Cells were fixed 8 h later and stained with anti-M2 (Mab E10; red) and DAPI (blue) followed by imaging with confocal microscopy. Examples of optical sections are shown, either as merged 3-color images or the red, green, and blue channels alone (in grey scale). (A) 63x magnification. (B) 3x magnification of the same images shown in A. Brightness was adjusted for optimal clarity, with all images treated equally. CQ: chloroquine. (PDF) [file ppat.1007892.s007.pdf]
