# Supplementary material for: Dysregulation of M segment gene expression contributes to influenza A virus host restriction
Source: PLoS Pathog. 2019 Aug 15;15(8):e1007892. doi: 10.1371/journal.ppat.1007892 (PMC6695095; doi:10.1371/journal.ppat.1007892)
Supplement: S8 Fig — A549 cells were inoculated at a MOI of 5 PFU/cell with PR8 viruses encoding avian or human-derived M segments and incubated in the presence or absence of 60 μM chloroquine from 1 hpi. Cells were lysed with whole cell lysis buffer following 8 h incubation at 37°C. Western immunoblot of virus-infected A549 cells: (A) Vinculin expression was measured to allow normalization of viral protein levels. NP expression was measured to assess viral replication. Levels of M1 and M2 protein expression were assessed using an antibody (Mab E10) to a common epitope at the amino terminus of M1 and M2 proteins, allowing relative expression to be assessed. Levels of LC3B I and II were assessed using an antibody that detects both the precursor and the activated forms of LC3B protein. Data presented are representative Western immunoblots from three independent experiments. LC3B I protein and LC3B II protein (B, C) were normalized, quantitated and displayed as a percentage of total LC3B protein in the absence (B) or presence (C) of 60 μM chloroquine. Data presented in B-C, show the means with SD from three independent experiments. For each experiment, two replicate radiograms were quantitated. Statistical significance was assessed using ordinary two-way ANOVA. CQ: chloroquine. (PDF) [file ppat.1007892.s008.pdf]

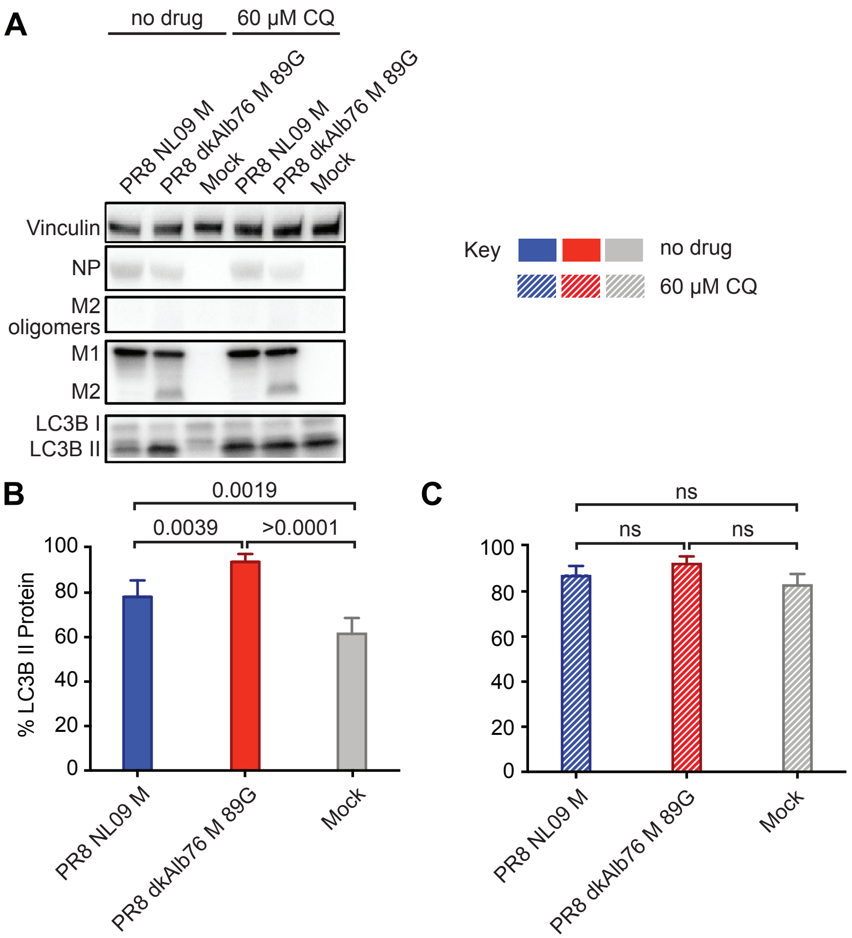

Supplementary Figure 8. Chloroquine treatment results in loss of activation of LC3B.

A549 cells were inoculated at MOI=5 with PR8 viruses possessing M segments from human or an avian host-derived strain and incubated in the presence or absences of 60 M chloroquine from 1 hpi. Cells were lysed with whole cell lysis buffer following 8 hours incubation at 37º C. Western blot of virus-infected A549 cells **(A)**. Vinculin expression was measured to allow normalization of viral protein levels. NP expression was measured to assess viral replication. Levels of M1 and M2 protein expression were assessed using an antibody (Mab E10) to a common epitope at the amino terminus of M1 and M2 proteins, allowing relative expression to be assessed. Levels of LC3B I and II were assessed using an antibody that detects both the precursor and the activated forms of LC3B protein. Data presented are representative Western blots from three independent experiments. LC3B I protein and LC3B II protein **(B, C)** were normalized, quantitated and displayed as a percentage of total LC3B protein in the absence **(B)** or presence **(C)** of 60 μM chloroquine. Data presented in **B-C**, show the means with SD from three independent experiments. For each experiment, two replicate radiograms were quantitated. Statistical significance was assessed using ordinary two-way ANOVA.
