# Supplementary material for: Dysregulation of M segment gene expression contributes to influenza A virus host restriction
Source: PLoS Pathog. 2019 Aug 15;15(8):e1007892. doi: 10.1371/journal.ppat.1007892 (PMC6695095; doi:10.1371/journal.ppat.1007892)
Supplement: S9 Fig — (A) Pandemic H1N1 influenza virus M1 and M2 proteins differ from the avian consensus sequences by 9 residues in M1 and 7 residues in M2. The pH1N1 amino acid identities are indicated in blue at their approximate positions within linear representations of M1 and M2 proteins. The pH1N1 M segment differs from the avian consensus by 8.3% at the nucleotide level. Here, pH1N1 RNA sequence is indicated by blue coloring. (B-D) Chimeric human-avian M segments were constructed in which only the non-synonymous changes in the avian consensus were introduced into the A/NL/602/09 (H1N1) M segment. (E) Avian consensus M1 and M2 proteins differ from the NL09 sequences by 9 residues in M1 and 7 residues in M2. The avian amino acid identities are indicated in red and avian RNA sequence is indicated by red coloring. (F-H) A second set of chimeric M segments was constructed in which the NL09 amino acid identities were introduced into the M segment of A/duck/Alberta/76 (H1N1) virus, yielding segments that encode avian consensus protein(s) but retain much of the nucleotide sequence of the NL09 M segment. (PDF) [file ppat.1007892.s009.pdf]

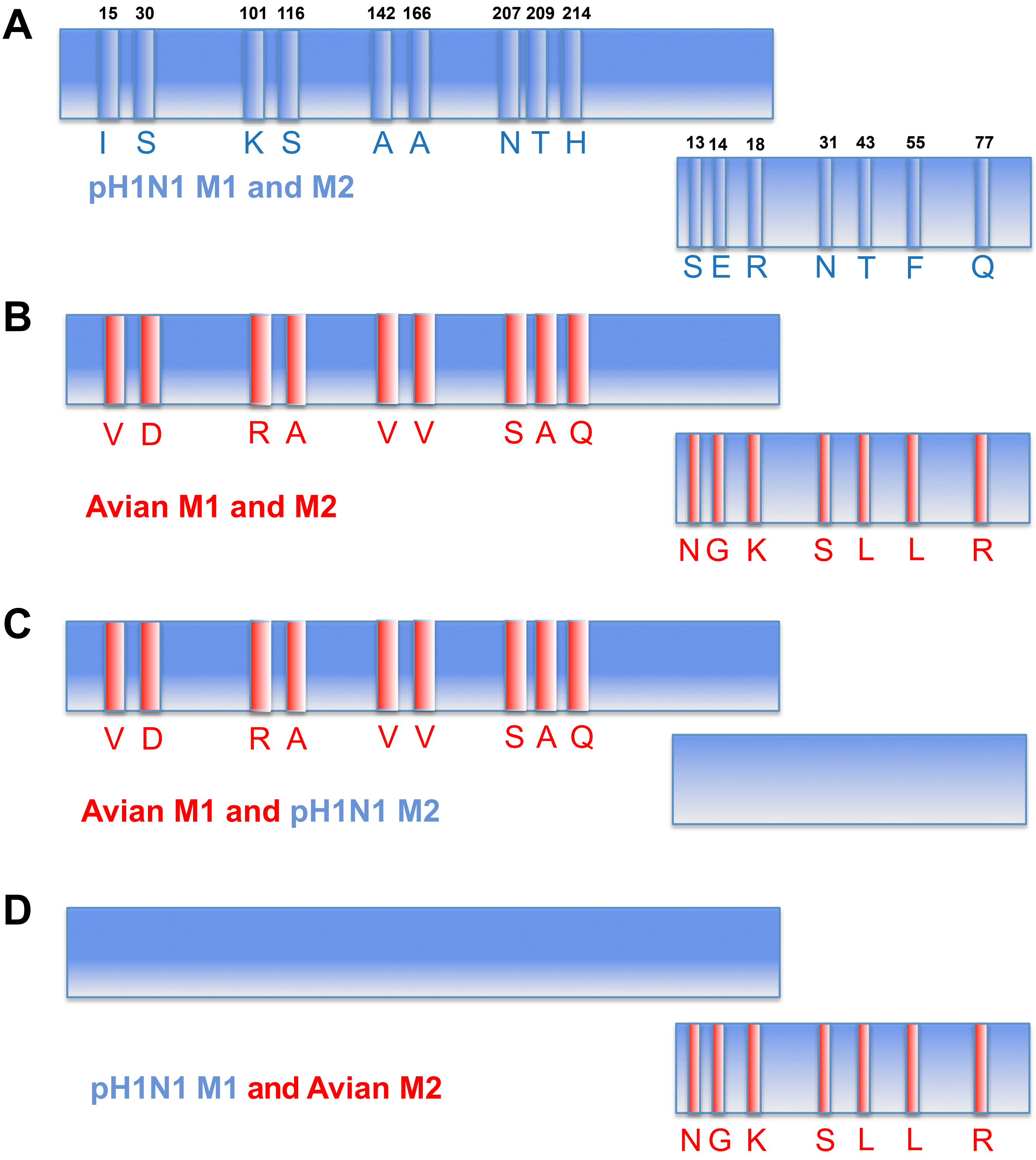
A
15 30 101 116 142 166 207 209 214
I S K S A A N T H
pH1N1 M1 and M2
13 14 18 31 43 55 77
S E R N T F Q
B
V D R A V V S A Q
Avian M1 and M2
N G K S L L R
C
V D R A V V S A Q
Avian M1 and pH1N1 M2
D
pH1N1 M1 and Avian M2
N G K S L L R

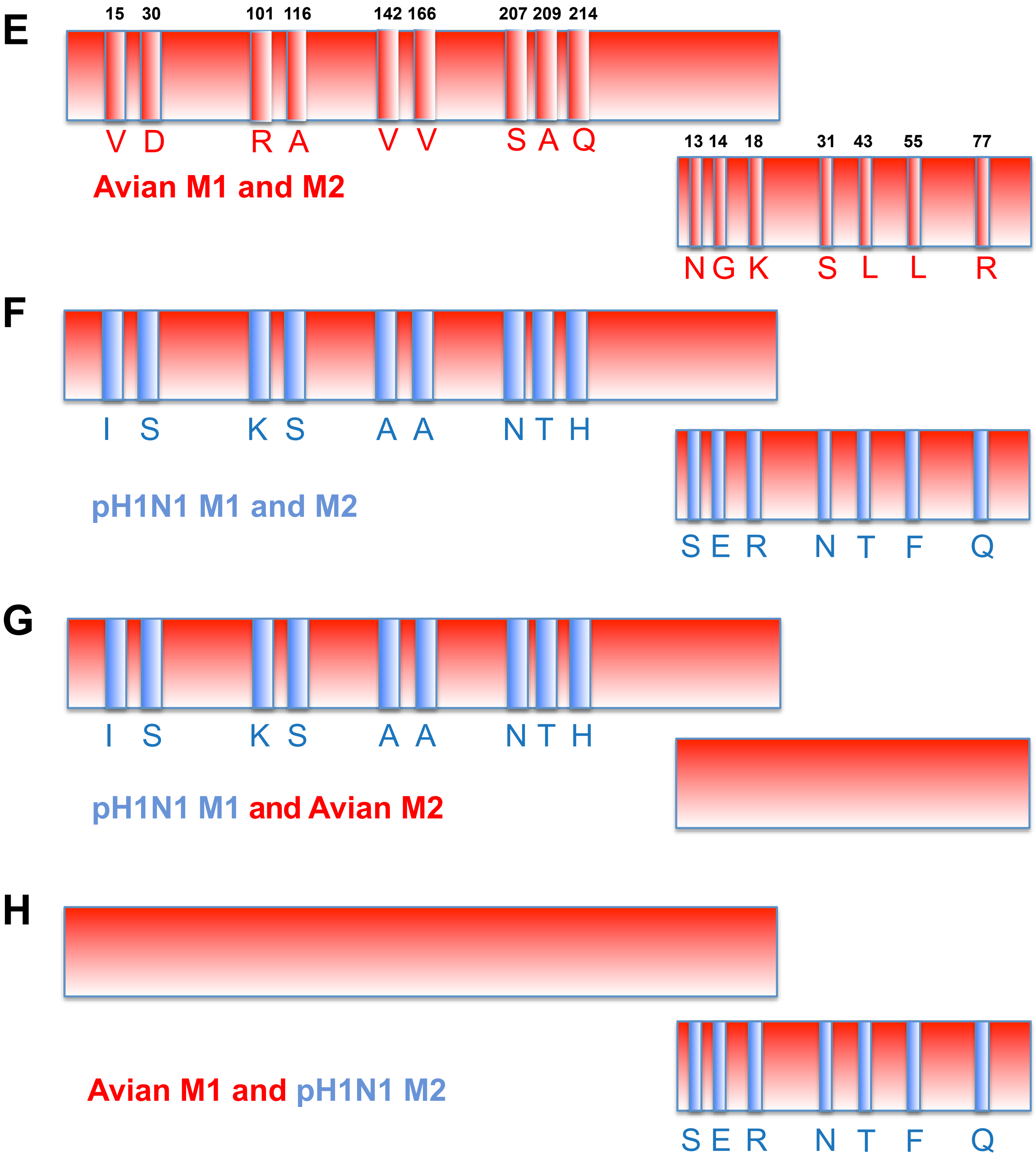

Supplementary Figure 9

Supplementary Figure 9. Schematic depicting chimeric M Segment RNAs

(A) Pandemic H1N1 influenza virus M1 and M2 proteins differ from the avian consensus sequences by 9 residues in M1 and 7 residues in M2. The pH1N1 amino acid identities are indicated in blue at their approximate positions within linear representations of M1 and M2 proteins. The pH1N1 M segment differs from the avian consensus by 8.3% at the nucleotide level. Here, pH1N1 RNA sequence is indicated by blue coloring. **(B-D)** Chimeric human-avian M segments were constructed in which only the non-synonymous changes in the avian consensus were introduced into the A/NL/602/09 (H1N1) M segment. **(E)** Avian consensus M1 and M2 proteins differ from the NL/09 sequences by 9 residues in M1 and 7 residues in M2. The avian amino acid identities are indicated in red and avian RNA sequence is indicated by red coloring. **(F-H)** A second set of chimeric M segments was constructed in which the NL/09 amino acid identities were introduced into the M segment of A/duck/Alberta/76 (H1N1) virus, yielding segments that encode avian consensus protein(s) but retain much of the nucleotide sequence of the NL/09 M segment.
