# Supplementary material for: Dysregulation of M segment gene expression contributes to influenza A virus host restriction
Source: PLoS Pathog. 2019 Aug 15;15(8):e1007892. doi: 10.1371/journal.ppat.1007892 (PMC6695095; doi:10.1371/journal.ppat.1007892)
Supplement: S10 Fig — 293T cells were transduced with GFP-LC3 protein and inoculated 24 h later with the indicated IAVs, encoding avian, human or chimeric M segments, at a MOI of 5 PFU/cell. Cells were fixed 12 h later and stained with anti-M2 (Mab E10; red) and DAPI (blue) followed by imaging with confocal microscopy. Examples of optical sections are shown, either as merged 3-color images or the red, green, and blue channels alone (in grey scale). 3x magnification of 63x images are shown. Brightness was adjusted for optimal clarity, with all images treated equally. (PDF) [file ppat.1007892.s010.pdf]

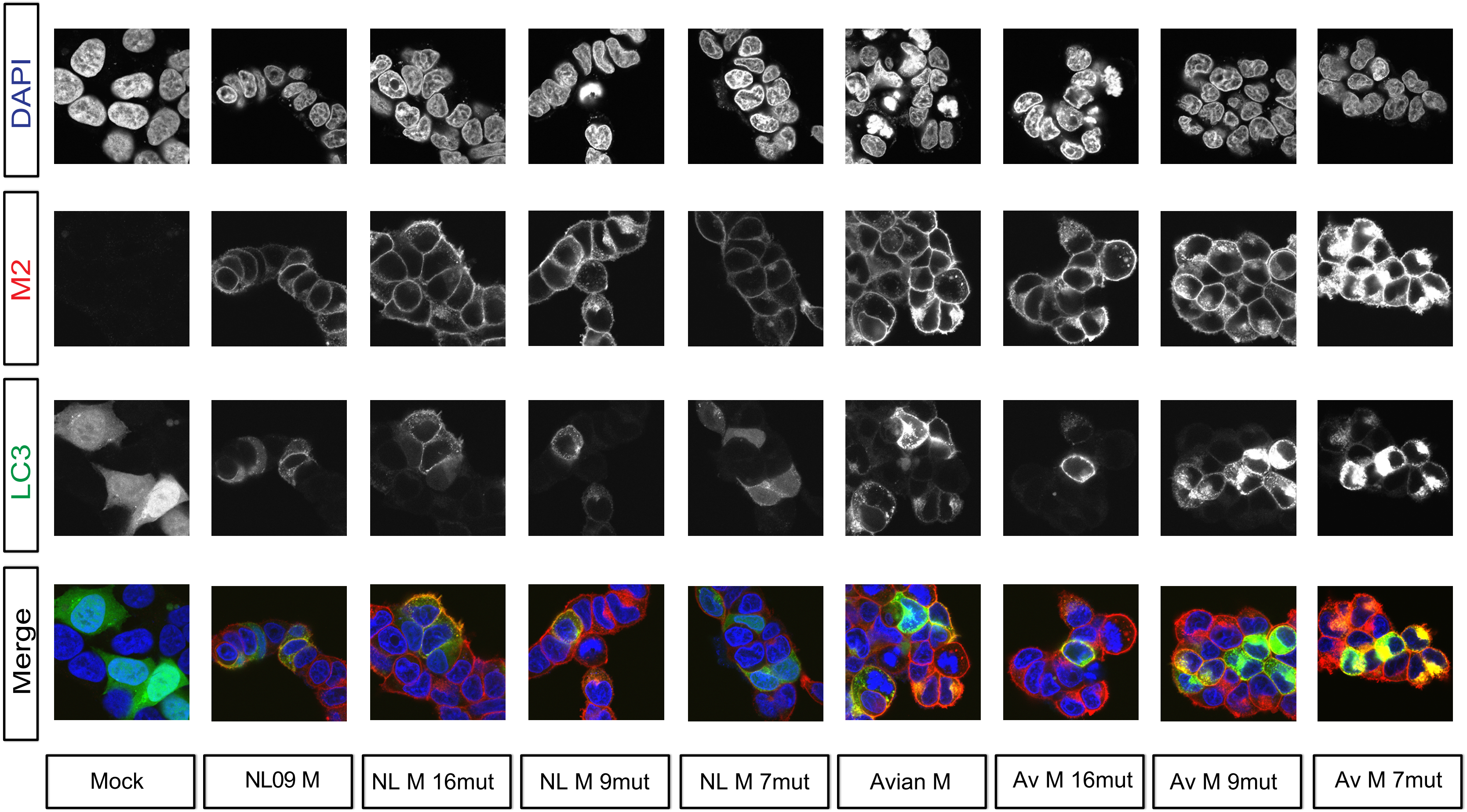

Supplementary Figure 10. Visualization of LC3 and M2 co-localization in 293T cells infected with viruses carrying avian-human chimeric M segments.

293T cells were transduced with GFP-LC3 protein and inoculated 24 h later with the indicated IAVs, encoding avian, human or chimeric M segments, at an MOI of 5 PFU/cell. Cells were fixed 12 h later and stained with anti-M2 (Mab E10; red) and DAPI (blue) followed by imaging with confocal microscopy. Examples of optical sections are shown, either as merged 3-color images or the red, green, and blue channels alone (in grey scale). 3x magnification of 63x images are shown. Brightness was adjusted for optimal clarity, with all images treated equally.
