# Supplementary material for: Dysregulation of M segment gene expression contributes to influenza A virus host restriction
Source: PLoS Pathog. 2019 Aug 15;15(8):e1007892. doi: 10.1371/journal.ppat.1007892 (PMC6695095; doi:10.1371/journal.ppat.1007892)
Supplement: S11 Fig — A549 cells were inoculated at a MOI of 5 PFU/cell with PR8 NL09 M virus and PR8 Av M virus, along with six PR8-based viruses with chimeric M segments. Cells were incubated at 37°C for 8 h and then lysed with a RNeasy Kit. (A) RT primer extension radiogram of virus-infected A549 cells. 5S rRNA levels were measured to allow normalization of viral RNA. Segment 7 vRNA expression was measured to assess viral replication. (B) Levels of mRNA7, (C) mRNA10, and (D) mRNA11 were quantitated and displayed as a percentage of total M gene expressed mRNA. Graphs in B-D show the means with SD from three independent experiments. For each experiment, two replicate radiograms were quantitated. Statistical significance was assessed using ordinary one-way ANOVA. (PDF) [file ppat.1007892.s011.pdf]

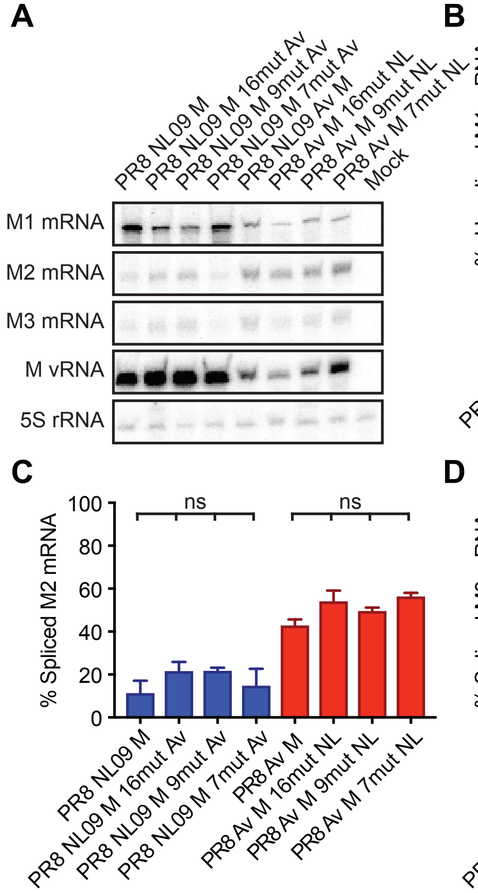

Supplementary Figure 11. Levels of mRNA7 (encoding M1) and mRNA10 (encoding M2) transcripts in A549 cells infected with viruses carrying human, avian or chimeric M segments.

A549 cells were inoculated at an MOI=5 with PR8 NL09 M virus and PR8 Av M virus, along with six PR8-based viruses with chimeric M segments. Cells were incubated at 37° C for 8 hours and then lysed with an RNeasy Kit. **(A)** RT primer extension radiogram of virus-infected A549 cells. 5S rRNA levels were measured to allow normalization of viral RNA. Segment 7 vRNA expression was measured to assess viral replication. Levels of mRNA7 **(B)**, mRNA10 **(C)**, and mRNA11 **(D)** were quantitated and displayed as a percentage of total M gene expressed mRNA. Graphs in B-D show the means with SD from three independent experiments. For each experiment, two replicate radiograms were quantitated. Statistical significance was assessed using ordinary one-way ANOVA.
