# Supplementary material for: Dysregulation of M segment gene expression contributes to influenza A virus host restriction
Source: PLoS Pathog. 2019 Aug 15;15(8):e1007892. doi: 10.1371/journal.ppat.1007892 (PMC6695095; doi:10.1371/journal.ppat.1007892)
Supplement: S12 Fig — Groups of four guinea pigs were inoculated with 10 PFU of each chimeric M-encoding virus, as indicated. Graphs show individual titers obtained from animals used in three independent experiments. Virus replication in nasal wash of inoculated animals was measured by plaque titration at days 2, 4, 6, and 8 post-infection and the titers at each time point were plotted (dotted lines). The differences between PR8 NL09 M 7 mut Av and avian M 9 mut NL encoding viruses were considered significant. Statistical significance in kinetics of growth was determined by assessing the interaction of time and virus using repeated measures, two-way, multiple comparisons ANOVA on mean values, with Bonferroni correction applied to account for comparison of a limited no of means. (PDF) [file ppat.1007892.s012.pdf]

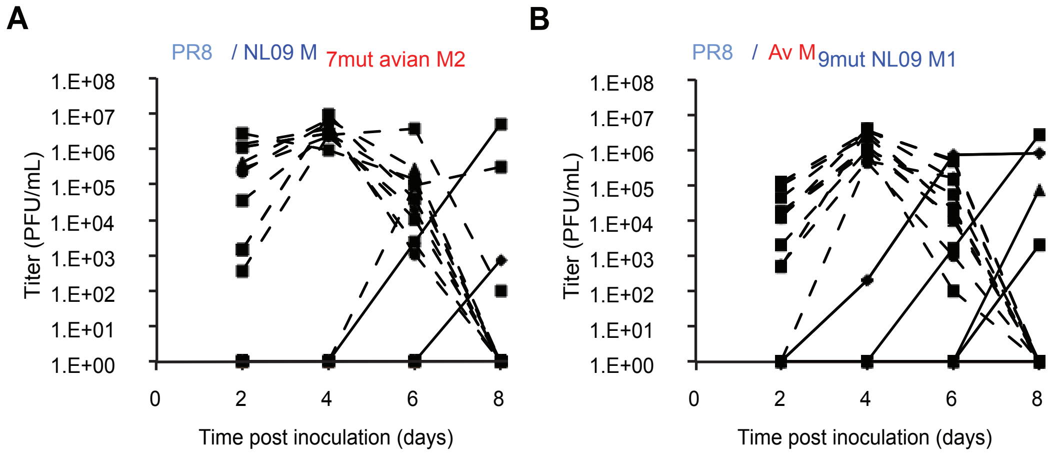


A v B: P=0.0083

**Supplementary Figure 12. High Expression of M2 Protein Reduces Kinetics of Replication of PR8-Based Viruses in Guinea Pigs.**

Groups of four guinea pigs were inoculated with 10 PFU of each chimeric M-encoding virus, as indicated. Graphs show individual titers obtained from animals used in three independent experiments. Virus replication in nasal wash of inoculated animals was measured by plaque titration at days 2, 4, 6, and 8 post-infection and the titers at each time point were plotted (dotted lines). The differences between PR8 NL09 M 7 mut Av and avian M 9 mut NL encoding viruses were considered significant. Statistical significance in kinetics of growth was determined by assessing the interaction of time and virus using repeated measures, two way, multiple comparisons ANOVA on mean values, with Bonferroni correction applied to account for comparison of a limited no of means.
