# Supplementary material for: Dysregulation of M segment gene expression contributes to influenza A virus host restriction
Source: PLoS Pathog. 2019 Aug 15;15(8):e1007892. doi: 10.1371/journal.ppat.1007892 (PMC6695095; doi:10.1371/journal.ppat.1007892)
Supplement: S13 Fig — 293T cells were inoculated with the indicated IAVs, encoding avian-, human- or PR8-derived M segments, at a MOI of 5 PFU/cell, or treated with 60 μM chloroquine. Cells were fixed 8 h later and stained with anti-M2 (Mab E10; red) and DAPI (blue) followed by imaging with confocal microscopy. Examples of optical sections are shown, either as merged 2-color images or the red and blue channels alone (in grey scale). 63x magnification with 3x optical zoom. Brightness was adjusted for optimal clarity, with all images treated equally. CQ: chloroquine. (PDF) [file ppat.1007892.s013.pdf]

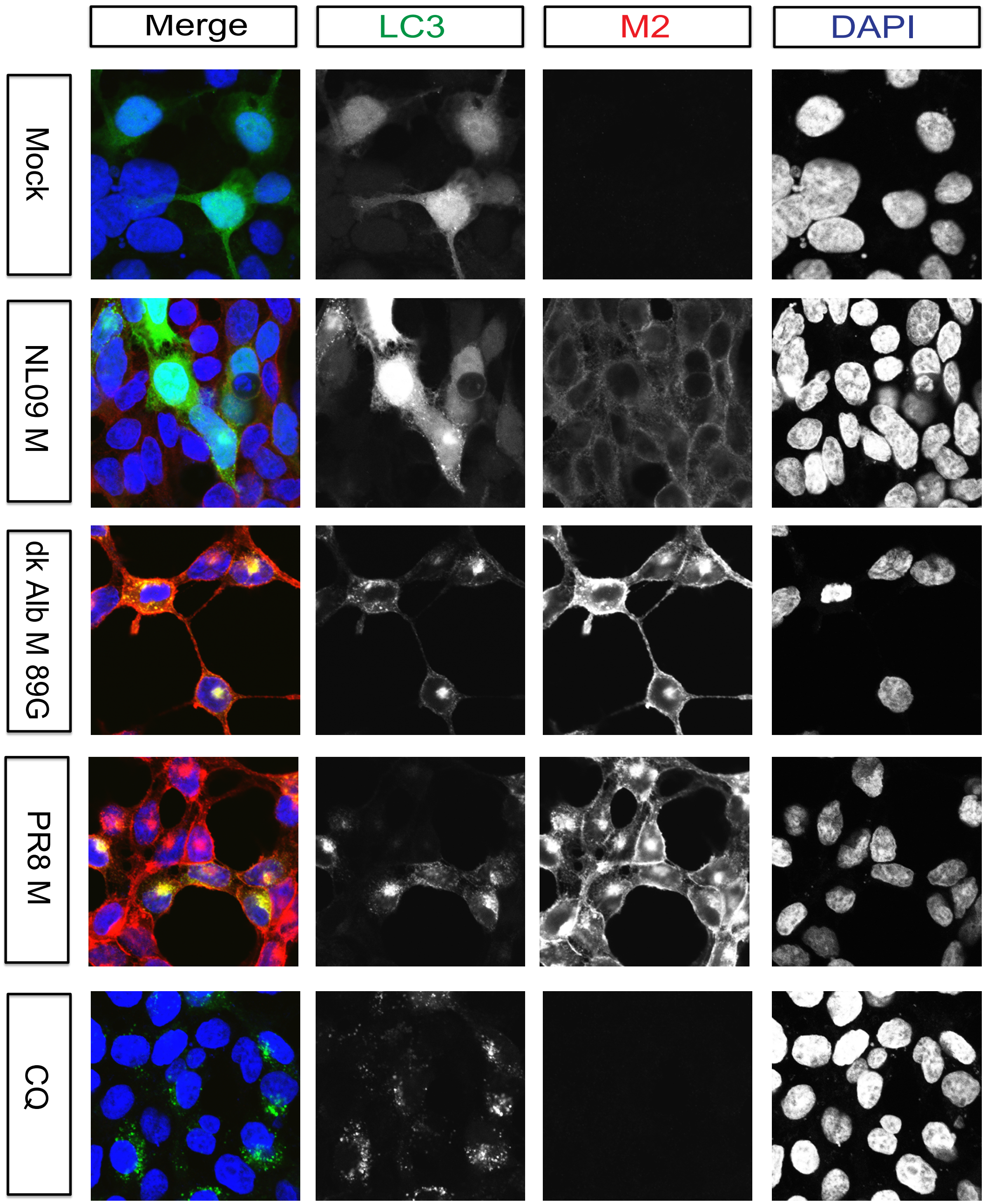

Supplementary Figure 13. PR8 M2 protein is over-expressed and partly localized in perinuclear vesicles in 293T cells

293T cells were inoculated with the indicated IAVs, encoding avian, human or PR8 derived M segments, at an MOI of 5 PFU/cell, or treated with 60uM chloroquine. Cells were fixed 8 h later and stained with anti-M2 (Mab E10; red) and DAPI (blue) followed by imaging with confocal microscopy. Examples of optical sections are shown, either as merged 2-color images or the red and blue channels alone (in grey scale). 63x magnification with 3x optical zoom. Brightness was adjusted for optimal clarity, with all images treated equally.
