# Supplementary material for: Dysregulation of M segment gene expression contributes to influenza A virus host restriction
Source: PLoS Pathog. 2019 Aug 15;15(8):e1007892. doi: 10.1371/journal.ppat.1007892 (PMC6695095; doi:10.1371/journal.ppat.1007892)
Supplement: S1 Table — The sequences analyzed to generate avian consensus sequences for M1 and M2 proteins are summarized. (DOCX) [file ppat.1007892.s014.docx]

**S1 Table. IAV M1 and M2 consensus sequences from virus strains circulating in wild waterfowl hosts are highly conserved**

| Subtype | No of Sequences acquired to generate consensus M1/M2 | M1 changes | M2 changes | % M1 Amino acid identity to avian consensus^1,2^ | % M2 Amino acid identity to avian consensus^1,2^ |
| --- | --- | --- | --- | --- | --- |
| H1Nx | 152/151 | None | None | 100 | 100 |
| H2Nx | 167/160 | None | None | 100 | 100 |
| H3Nx | 345/321 | None | None | 100 | 100 |
| H4Nx | 225/187 | None | None | 100 | 100 |
| H5Nx | 224/181 | None | None | 100 | 100 |
| H6Nx | 371/315 | None | None | 100 | 100 |
| H7Nx | 267/252 | None | None | 100 | 100 |
| H8Nx | 10/10 | None | None | 100 | 100 |
| H9Nx | 186/108 | V15I; R101K; I107M; F144L; V166A; L234I. | K17R; L55F. | 97.6 (6aa) | 97.9 (2aa) |
| H10Nx | 196/92 | None | None | 100 | 100 |
| H11Nx | 186/182 | None | None | 100 | 100 |
| H12Nx | 43/42 | None | None | 100 | 100 |
| H13Nx | 28/28 | None | P10H; N13S; K17R; R77Q; Q80K. | 100 | 94.8 (5aa) |
| H14Nx | 4/4 | None | None | 100 | 100 |
| H15Nx | 7/7 | None | None | 100 | 100 |
| H16Nx | 10/10 | None | P10H; N13S; K17R; R61S; R77Q; Q80K. | 100 | 93.8 (6aa) |
| pH1N1 | 788/730 | V15I; D30S; R101K; A116S; V142A; V166A; S207N; A209T; Q214H. | N13S; G14E; K17R; S31N; L43T; L55F R77Q. | 96.4 (9 aa) | 92.8 (7aa) |

^1^ Avian consensus sequences were determined from sequences collected between the years 1970 and 2000 and deposited in the Genbank database.

^2^Sequences were aligned by Clustal W algorithm using Megalign software (DNASTAR).
